# Using antenatal care as a platform for malaria surveillance data collection: study protocol

**DOI:** 10.1186/s12936-023-04521-6

**Published:** 2023-03-17

**Authors:** Julie R. Gutman, Julia Nanteza Mwesigwa, Kyra Arnett, Chabu Kangale, Sijenunu Aaron, Dele Babarinde, Julie Buekens, Baltazar Candrinho, Siaka Debe, Peder Digre, Mary Drake, Adama Gansané, Christelle Gogue, Kevin S. Griffith, Joseph Hicks, Réné Kinda, Hannah Koenker, Ruth Lemwayi, Anna Munsey, Emmanuel Obi, Aurore Ogouyèmi-Hounto, Okefu Oyale Okoko, Faustin Onikpo, Ali Onoja, Travis Porter, Binete Savaio, Kenzie Tynuv, Perpetua Uhomoibhi, Joseph Wagman, Katherine Wolf, Rose Zulliger, Patrick Walker, John M. Miller, Molly Robertson

**Affiliations:** 1 Malaria Branch, US Centers for Disease Control and Prevention, Atlanta, GA USA; 2 PATH, Kampala, Uganda; 3 PATH, Seattle, WA USA; 4 PATH, Lusaka, Zambia; 5 National Malaria Control Program, Dodoma, Tanzania; 6 Ibolda Health International Ltd, Abuja, Nigeria; 7 MCD Global Health, Silver Spring, MD USA; 8 Programa Nacional de Controlo da Malária, Maputo, Mozambique; 9 Centre National de Recherche Et de Formation Sur Le Paludisme, Ouagadougou, Burkina Faso; 10 Jhpiego, Dar Es Salaam, Tanzania; 11 PATH, Washington, DC USA; 12 US President’s Malaria Initiative, US Agency for International Development, Washington, DC USA; 13 Imperial College London, London, UK; 14 Tropical Health, Baltimore, MD USA; 15 National Malaria Elimination Program, Abuja, Nigeria; 16 Faculté Des Sciences de La Santé/Université d’Abomey Calavi, Godomey, Benin; 17 PATH, Maputo, Mozambique; 18 Jhpiego, Baltimore, MD USA; 19 The Global Fund to Fight AIDS, Tuberculosis and Malaria, Geneva, Switzerland

**Keywords:** ANC-based surveillance, Benin, Burkina Faso, Malaria, Mozambique, Nigeria, Tanzania, Zambia

## Abstract

**Background:**

While many malaria-endemic countries have health management information systems that can measure and report malaria trends in a timely manner, these routine systems have limitations. Periodic community cross-sectional household surveys are used to estimate malaria prevalence and intervention coverage but lack geographic granularity and are resource intensive. Incorporating malaria testing for all women at their first antenatal care (ANC) visit (i.e., ANC1) could provide a more timely and granular source of data for monitoring trends in malaria burden and intervention coverage. This article describes a protocol designed to assess if ANC-based surveillance could be a pragmatic tool to monitor malaria.

**Methods:**

This is an observational, cross-sectional study conducted in Benin, Burkina Faso, Mozambique, Nigeria, Tanzania, and Zambia. Pregnant women attending ANC1 in selected health facilities will be tested for malaria infection by rapid diagnostic test and administered a brief questionnaire to capture key indicators of malaria control intervention coverage and care-seeking behaviour. In each location, contemporaneous cross-sectional household surveys will be leveraged to assess correlations between estimates obtained using each method, and the use of ANC data as a tool to track trends in malaria burden and intervention coverage will be validated.

**Results:**

This study will assess malaria prevalence at ANC1 aggregated at health facility and district levels, and by gravidity relative to current pregnancy (i.e., gravida 1, gravida 2, and gravida 3 +). ANC1 malaria prevalence will be presented as monthly trends. Additionally, correlation between ANC1 and household survey–derived estimates of malaria prevalence, bed net ownership and use, and care-seeking will be assessed.

**Conclusion:**

ANC1-based surveillance has the potential to provide a cost-effective, localized measure of malaria prevalence that is representative of the general population and useful for tracking monthly changes in parasite prevalence, as well as providing population-representative estimates of intervention coverage and care-seeking behavior. This study will evaluate the representativeness of these measures and collect information on operational feasibility, usefulness for programmatic decision-making, and potential for scale-up of malaria ANC1 surveillance.

**Supplementary Information:**

The online version contains supplementary material available at 10.1186/s12936-023-04521-6.

## Background

Malaria continues to be a significant cause of morbidity and mortality worldwide, and progress toward malaria control and elimination targets in many countries has now stalled due to a range of complex issues, including increased insecticide resistance, suboptimal control strategies, weak public health systems, reduced funding, and shifting global health priorities [1]. There were an estimated 247 million malaria cases in 2021, an increase from 227 million cases in 2019 [2, 3].

Health surveillance systems continuously monitor disease trends that inform the planning, implementation, and evaluation of prevention and control programs, as well as the allocation of health resources. The World Health Organization’s *Global Technical Strategy for Malaria 2016–2030* describes surveillance as a key pillar of national and subnational strategies, necessary at all levels of malaria burden to achieve elimination [4]. However, current surveillance systems are costly and/or prone to error. Malaria-endemic countries have implemented standardized, electronic-based health management information systems (HMISs) on platforms such as the District Health Information Software 2 (DHIS2), which can provide real-time, monthly health facility data to measure trends in clinical malaria from outpatient department visits. However, timeliness and quality can be limited by the fact that the data are often collected on paper registers and must be tallied and uploaded to the DHIS2 system. Another key limitation is that these data are collected from public health systems and are subject to patient care-seeking behavior, and thus may not be representative of malaria transmission in the broader community [5]. Further, calculating accurate incidence measurements at the most granular level requires accompanying information about the population at the level of the health facility catchment area. In addition, although other country data platforms may exist, HMISs themselves do not currently collect information on intervention coverage or use and, despite recent improvements, data quality remains a limitation of these systems in many contexts [2]. In combination, these limitations mean that in the *World Malaria Report 2021*, the World Health Organization (WHO) deemed that the quality of surveillance data did not permit a robust estimate of disease burden from the number of reported cases for 30 malaria-endemic countries, all of which were in Africa [2]. Moreover, there are currently no routine indicators with which to assess trends in exposure to malaria in pregnancy, a key driver of many adverse pregnancy and birth outcomes, and a substantial contributor to overall malaria burden [6, 7], nor is there an indicator to assess the prevalence of asymptomatic malaria infections that contribute significantly to transmission.

Household cross-sectional surveys such as demographic health surveys and malaria indicator surveys are used to measure infection prevalence among both asymptomatic and symptomatic persons combined (and specifically, among children under 5 years of age in most surveys), coverage of malaria control interventions, and care-seeking behaviours in the general population to help provide accurate estimates of these indicators. They are also the basis for many geostatistical models of malaria burden, particularly in countries lacking sufficiently robust routine data, and often serve as a cornerstone of subnational stratification and tailoring exercises, which inform strategic plan and funding application development [8]. However, household surveys are rarely powered sufficiently to detect sub-provincial differences in these outcomes and are typically implemented every 2 to 5 years, making clear longitudinal trends difficult to assess. Additionally, in comparison to routine HMIS data, cross-sectional surveys are less granular, are unable to monitor short-term changes in malaria burden, and have limited use for real-time decision-making or subnational stratification.

Integrating malaria surveillance into routine antenatal care (ANC) visits has been proposed as a strategy to obtain representative data able to monitor population trends in malaria burden and intervention coverage in real time [9]. Continuous sentinel surveillance of pregnant women by testing them at their first scheduled antenatal care visit (ANC1) before they have received intermittent preventive treatment (IPTp) could be used to measure symptomatic and asymptomatic infections, provide data on spatial and temporal trends, add robustness to subnational geostatistical models, identify geographical areas with higher-than-average transmission intensity (“hot spots”), evaluate sustained changes in transmission following new interventions, and provide an early warning of increasing transmission. Women at ANC clinics could also participate in surveys about coverage, access, and use of malaria preventive tools, potentially allowing rapid assessments of intervention coverage, use, and progress in programme implementation, all with a higher degree of granularity and temporal resolution than household surveys.

Studies indicate that pregnant women attending ANC1 would be highly representative of the overall population of pregnant women where ANC attendance is high, and potentially representative of malaria prevalence in the broader population [9–12]. ANC coverage is high in sub-Saharan Africa, with an average of 72% of pregnant women attending at least one ANC visit in countries with moderate-to-high malaria transmission [3]. Monthly ANC attendance remains consistent over time, with less seasonal variation in attendance as compared to febrile visits to the outpatient department. Additionally, there is less correlation between the reason for attendance (ANC) and the outcome of interest (malaria) as compared to outpatient department attendance, where a large proportion of people present for evaluation of fever. Prevalence estimates using ANC1-based surveillance of all women regardless of symptoms would not be affected by rates of non-malarial febrile illness [13], which are likely to influence malaria test positivity rates obtained only from individuals with fever [14].

It has also been demonstrated that malaria prevalence among pregnant women correlates with the prevalence among children under 5 years of age and other populations [10–12, 15]. A systematic review and meta-analysis comparing prevalence of malaria infection in pregnant women to prevalence in children under five in the same region and during the same period showed a strong correlation between groups, with the strongest correlation between children under five and primigravid women [9]. In Tanzania, several studies have demonstrated a correlation between malaria prevalence in pregnant women attending ANC visits and other sources of prevalence estimates. In one study, data from 8 million pregnant women attending routine nationwide malaria screening at ANC1 before their first dose of IPTp were compared to data from 15,000 children under five, the latter being the total data from population-based household surveys in Tanzania in the equivalent time period. A strong positive correlation was found between the two indicators, with ANC data able to identify temporal and district-level trends well beyond the power of the survey data [12]. This suggests that ANC-based surveillance could be a highly cost-effective way to interpolate between survey data in both time and space toward the resolution needed for timely and tailored strategic action and allocation of resources [15].

While women attending ANC1 could be a pragmatic sentinel population for deriving granular and timely information on malaria burden, this measurement platform has not been thoroughly evaluated in multiple countries against current gold standard cross-sectional household surveys. This study leverages ongoing cross-sectional household surveys and contemporaneous ANC1 data collection to assess correlations and validate the use of ANC1 data to track monthly trends in malaria burden and intervention coverage, such as coverage and use of insecticide-treated nets and care seeking for febrile illness. In addition, where population denominator data are available, a secondary analysis will be conducted to look at the relationship between ANC1 malaria prevalence and malaria case incidence data derived from DHIS2.

This protocol provides a methodology and questionnaire for other national malaria control programs that may be interested in utilizing ANC1 surveillance to understand the pros and cons of using these data.

## Objectives

The overall study objective is to assess the correlation between monthly malaria prevalence estimates obtained from ANC1-based surveillance and from cross-sectional household surveys assessing malaria prevalence among children under 5 years of age to validate the ANC1 surveillance approach as (a) representative of the metric of malaria prevalence reported by common household surveys and (b) useful for monitoring general trends in malaria burden and coverage of malaria control interventions over time.

## Study outcomes

### Primary outcome

1. Monthly prevalence of rapid diagnostic test (RDT)-confirmed malaria infections among women at ANC1 (asymptomatic and symptomatic).
2. Correlation at health facility and district level between malaria prevalence at the ANC1 (monthly or quarterly) and among children tested during cross-sectional household surveys.

### Secondary outcomes

1. Comparison of ANC1 data to cross-sectional household survey data to ascertain whether pregnant women attending ANC1 can be a sentinel population for estimating, both overall and by gravidity:
  - Bed net ownership and use the previous night.
  - Population access to bed nets (proportion of households achieving one net for every two people).
  - Care-seeking behaviour for malaria.
2. Reported acceptability and feasibility of ANC1 malaria surveillance to women and providers.
3. Relationship between ANC1 malaria prevalence and malaria case incidence derived from DHIS2 (where population denominator data are available).

## Methods

### Study design

This is a multi-country, observational, serial cross-sectional study with data from pregnant women attending ANC1 aggregated monthly. These data will be compared to similar survey questions (adapted from standard demographic health survey and malaria indicator survey questions) administered to households as part of cross-sectional surveys being conducted for evaluation of a number of different interventions (ClinicalTrials.gov Identifier: NCT04716387, NCT04157894, NCT04148690). Malaria prevalence by RDT among children under 5 years of age collected during the household surveys will also be compared to the prevalence among pregnant women at ANC1 in the same or a similar time period.

### Study population

All pregnant women of legal age and emancipated minors attending ANC1 at selected health facilities in Benin, Burkina Faso, Mozambique, Nigeria, Tanzania, and Zambia are approached for enrollment (Table 1). In Mozambique and Nigeria only, pregnant women below the legal age are considered eligible with the consent of a legal guardian. Pregnant women with symptoms of severe disease as determined by the clinician at the ANC clinic are excluded and referred for urgent case management. Children under 5 years of age whose parents respond to the cross-sectional household survey and provide consent for malaria testing of their children are also included.

**Table 1:** Study site, participant, and survey details

Country	Study location	Number of study health facilities collecting ANC1 data	Age of women enrolled (years)	Study collecting cross-sectional household survey data	Methodology for identification of target health facility (HF) catchments	Sample size for cross-sectional household survey
Benin	Atlantique Department	40	15–49	Group Antenatal Care study	HFs with 30–120 first ANC visits per month that were easily accessible throughout majority of the year were selected, catchment areas were identified with the assistance of the HF staff	2,520 (of which 1,320 were selected from women with a completed pregnancy in the past year), twice
Burkina Faso	Banfora District	7	20–49	New Nets Project	HFs with > 20 first ANC visits per month on average in DHIS2 from 2017	190, annually
	Gaoua District	7				190, annually
	Orodara District	7				190, annually
Mozambique	Changara District	7	18–49	New Nets Project	HFs with > 20 first ANC visits per month on average in DHIS2 from 2017 – 2019	420, annually
	Guro District	7				420, annually
	Chemba District	7				420, annually
Nigeria	Asa local government area	10	18–49	New Nets Project	By LGA, ten HFs with the most first ANC visits per month reported in DHIS2 in 2019	420, annually
	Ejigbo local government area	10				420, annually
	Ife North local government area	10				420, annually
	Moro local government area	10	14–49			420, annually
Tanzania	Geita Region	40	15–49	Group Antenatal Care study	HFs with 30–120 first ANC visits per month that were easily accessible throughout majority of the year were selected, catchment areas were identified with the assistance of the HF staff	2,320 (1,120 were selected from women with a completed pregnancy in the past year), twice
Zambia	Chadiza District	21	15–49	Proactive community case management	All HFs in the district were included and catchment area boundaries were defined by the Ministry of Health	2178, twice

### Study settings and location

This study is leveraging several planned intervention studies, including cross-sectional household surveys (modified malaria indicator surveys) at the district or village level that collect parasite prevalence data among children under 5 years (and other age groups as well, depending on country-specific epidemiology and national malaria control programme priorities). In Burkina Faso, Mozambique, and Nigeria, study activities are aligned with surveys from the New Nets Project, an observational quasi-experimental study that is evaluating the cost-effectiveness of various types of insecticide-treated nets [16]. During the New Nets Project, annual cross-sectional surveys are conducted from 2019 to 2022, including assessments of malaria prevalence, and corresponding ANC1-based surveillance occurs in a subset of health facilities in corresponding districts. Additionally, studies assessing the impact of group ANC are planned in the catchment areas of 40 health facilities each in Geita Region, Tanzania, and Atlantique Department, Benin, with corresponding cross-sectional surveys occurring as the primary method of evaluating the impact of the group ANC intervention. Due to the COVID-19 pandemic, the group intervention was stopped early in Tanzania, but the ANC1-based surveillance assessment and cross-sectional surveys continue. Cross-sectional surveys are conducted at study baseline and endline (November 2019 and June 2021 in Tanzania, February/March 2021 and September/October 2022 in Benin). In Chadiza, Zambia, this ANC1-based surveillance pilot leverages a study assessing the impact of proactive community case management in 21 health facility catchments, with surveys conducted in May 2021 and May 2023.

### Health facility selection

Health facilities and their catchment areas are selected for participation in the parent studies according to the needs of each study; of these, health facilities with a minimum of 20 ANC1 per month are selected for co-inclusion in this ANC surveillance pilot. In some studies, all eligible health facilities are included, while in others a subset of eligible facilities are randomly selected for inclusion. Two activities are integrated into routine ANC1 consultations: malaria testing of eligible participants regardless of symptoms using an RDT, and administration of a questionnaire to collect data on participant demographics, gravidity, insecticide-treated net ownership and use, and care-seeking behaviour. For each facility, the corresponding catchment villages were identified in collaboration with the health facility workers. From among all the villages in the health facility catchment area, 1–2 villages or enumeration areas were selected for household sampling for the cross-sectional survey proportional to population size. The selected areas were mapped and respondents randomly selected as indicated by the parent study conducting the cross sectional survey.

### ANC1 screening and enrollment

All pregnant women attending ANC1 are tested for malaria infection. During group counselling sessions at initial ANC1 intake, women are informed of this pilot surveillance activity. Women are consented for this pilot surveillance activity individually prior to testing. The number of women refusing malaria testing is noted on a screening form. For consenting women, a malaria RDT is administered concurrently with other routine ANC testing, using blood from the same finger prick. While awaiting the results from these tests, the short ANC surveillance questionnaire (Additional file 1) is administered by a trained study team or staff member, and the RDT result recorded when available. Women have the option of responding to the questionnaire even if they declined the RDT. During a regular ANC visit, the nurse asks questions to determine a woman’s age, gravidity, and gestational age. These data are recorded in the ANC registers and copied into the corresponding study questionnaires to avoid asking women the same questions twice. In Burkina Faso, Mozambique, and Zambia, a paid trained field worker assists with recruitment and administering the questionnaire while in the other countries, this task is completed by clinic staff.

### Blood sample collection

As stated above, finger-prick capillary blood samples are collected for malaria diagnosis using malaria RDTs. The RDT used for both ANC1 surveillance and the cross-sectional household surveys is determined by national procurement protocols. For ANC1 surveillance, the blood used for malaria testing is taken from the same finger prick that is used to collect blood for standard ANC testing. If positive, treatment is given to the women according to the national guidelines for malaria treatment.

### Sample size

A sample size of 88 people from a population of 1,000 people produces a two-sided 95% confidence interval with a precision (half-width) of 0.10 when the actual proportion is near 0.50. The sample size needed for a given confidence interval around a proportion is greatest for a proportion of 0.50. This is used to provide the most conservative assumption of sample size. The health facility sampling scheme is implemented across the different countries to provide a minimum of 88 women enrolled per month per sampling unit (i.e., health facility, district, or country), for a yearly total of 1,056 women per sampling unit. In cases where this sample size is not achieved in a single month at a given health facility, the data will be aggregated across facilities to provide a district/country estimate or across months to provide quarterly estimates to have sufficient sample size to provide an accurate estimate.

**Fig. 1:**
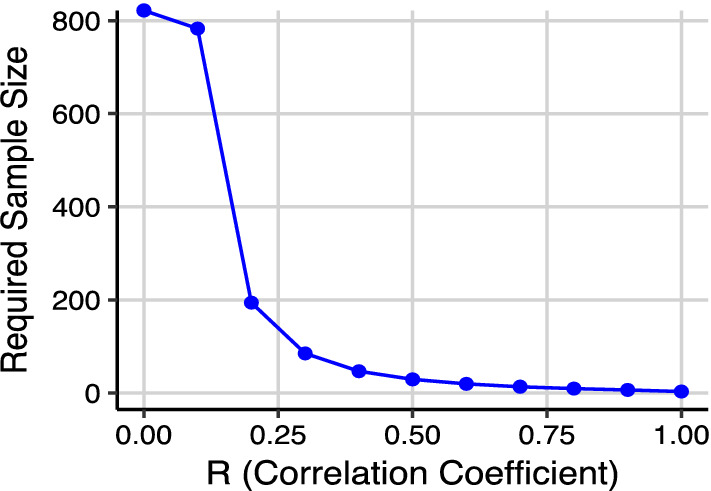
Sample size required for given correlation coefficient

To calculate the correlation between the facility-level data and community-level data, an estimated minimum of 20 facilities is needed to provide an accurate estimate of the correlation coefficient (R), using a two-tailed alpha test (alpha 0.05) and with 80% power, assuming an expected correlation coefficient of 0.6 or greater (Fig. 1). This is not feasible across all individual countries, thus using the combined data from multiple countries aimed to ensure sufficient sample sizes to assess the correlation.

### Data analysis and modelling

This study will summarize ANC1 participant demographic characteristics using descriptive statistics for age and gravidity by health facility and district. Key indicators will include:

- Total women attending ANC1, and proportion enrolled in ANC1 surveillance.
- Mean age of enrolled women (years; mean, 95% CI).
- Number enrolled by gravidity (n, %) by gravida 1, gravida 2, and gravida 3 + .

Prevalence is calculated as the number of women with positive RDT results divided by the total number of women tested [11]. Within each health facility and district, prevalence will be disaggregated by gravidity relative to the current pregnancy (i.e., first pregnancy [gravida 1], second pregnancy [gravida 2], and third or more pregnancy [gravida 3 +]). The monthly malaria prevalence data will be used to produce time series graphs, quarterly and/or annually, for correlation with cross-sectional survey estimates. In addition, where available, the proportion of RDT-positive women who are asymptomatic and symptomatic will be calculated.

To compare ANC1 malaria prevalence to prevalence from cross-sectional surveys within the same district, ANC1 malaria prevalence are obtained for the three months surrounding the community cross-sectional surveys (i.e., the month before the cross-sectional survey, the month of the survey, and the month directly following the cross-sectional survey). The relationship between malaria prevalence at ANC1 and in the community will be quantified using mixed-effects logistic regression, accounting for likely modifiers such as age, gravidity, transmission intensity, and transmission season, and adjusting for sampling effort in time and space. To quantify the incremental value of the ANC1 data, the ability of the ANC1 data to predict population prevalence using these models will be tested using leave-one-out cross-validation for each district and compared to alternative predictive models in the absence of any ANC1 data (e.g., extrapolating district-level trends from other districts sampled within the region). With support of national malaria control programmes and operational partners, district-level data on the temporal distribution of interventions within each region will be leveraged to assess the utility of ANC1 data to capture plausible trends in intervention impact. Similar analyses will be conducted to quantify the relationship between the cross-sectional surveys, the ANC1 data, reported case incidence, and the malaria test-positivity rate (obtained by dividing the number of positive malaria tests by the number of tests performed). In addition, the use of more heuristic measures such as the Kendall correlation coefficient, which provides a summary of the extent to which prevalence ranking of each district remains constant over time [12], will be used to assess the extent to which the choice of malaria metric influences operational decision-making.

Estimates of care-seeking behaviors and indicators for bed net ownership, use, and access will be determined for ANC1 data and compared to the corresponding indicators from the cross-sectional surveys. Net use and ownership (proportion of households that own at least one bed net and proportion of households with one net for every two people) will be aggregated monthly for each sampling unit/district and correlated with monthly ANC1 malaria prevalence to compare trends.

Once an optimal predictive model of community prevalence using ANC1 data has been obtained, this relationship will be incorporated within an existing, well-established mechanistic model of malaria transmission [17–19] in an attempt to provide a framework that can convert estimates of ANC1 malaria prevalence into continuous measures of community malaria transmission and burden with which to explore future intervention scenarios (analogous to a current framework that involves calibrating to cross-sectional data [20]).

### Data management

Data will be obtained from separately generated electronic or paper registers in each country. At each health facility, teams will be trained to conduct data quality assessments to monitor and improve data quality and completeness. Health facility–level ANC1 data from the DHIS2 system will include the monthly number of ANC attendees, the number tested for malaria at ANC1, and the number positive for malaria. Additional data will be collected on maternal age, gravidity, and insecticide-treated net ownership and utilization. Data on malaria prevalence among children will be obtained from representative, cross-sectional, household surveys as specified in the parent studies. Participants will be assigned a unique identifier number by the study, and will be identified only by this number in the dataset. No participant names or other information that would make the participant identifiable will be included.

### Consent

Individual written consent is obtained from women of legal age prior to testing and questionnaire administration during ANC1. In Mozambique and Nigeria, women under legal age are able to participate with the consent of their parent or legal guardian. Participants are told the general purpose, possible risks, and benefits of the ANC1 pilot surveillance activity in the local language. Participation is voluntary.

### Ethical clearance

Ethical clearance for the study was sought and obtained from the following institutional review boards:

- Comité d’Ethique de La Recherche de l’ISBA, Institut des Sciences Biomédicales Appliquées (Benin).
- PATH Research Ethics Committee (Burkina Faso, Mozambique, Zambia).
- Centre National de Recherche et de Formation sur le Paludisme (Burkina Faso).
- Comité d’Ethique pour la Recherche en Santé (Burkina Faso).
- Comité Institucional de Bioética para Saúde (Mozambique).
- National Health Research Ethics Committee of Nigeria (Nigeria).
- World Health Organization Research Ethics Review Committee (Nigeria).
- Medical Research Coordination Committee of the National Institute for Medical Research (Tanzania).
- National Health Research Ethics Committee (Zambia).

In addition, this activity was reviewed by CDC and was conducted consistent with applicable federal law and CDC policy.

## Discussion

This work builds on previous work assessing the validity of using pregnant women attending ANC1 as a sentinel population for monitoring malaria prevalence over time [10, 12]*.* In the present expanded observational study, ANC1-based surveillance collects additional data on indicators of bed net access and use and care-seeking behavior, information that is not collected in routine HMIS settings. The results generated from this study will be used to validate how reliably data collected during routine ANC visits can be used for malaria surveillance in malaria-endemic regions of sub-Saharan Africa.

### Public health importance of ANC-based malaria surveillance

In sub-Saharan Africa, approximately 72% of pregnant women attend ANC at least once during pregnancy [3], even in many rural areas where health care is not easily obtained. Malaria prevalence estimates from ANC1 attendees (before administration of IPTp) could, therefore, prove useful for monitoring changes in malaria transmission over time. ANC1-based surveillance could provide real-time, weekly or monthly data on malaria trends as well as information on access to control interventions such as ownership and use of insecticide-treated bed nets at the district and health facility level, which is sometimes missed in routine HMISs. Subtle changes in trends like early epidemics may be detectible, allowing more timely responses from malaria control programmes.

ANC1-based surveillance offers a basis for targeting malaria control interventions and resource allocation depending on local transmission levels [11]. As malaria transmission continues to decline and infections become more heterogeneous [21] in some settings, ANC1-based surveillance can be used as an adjunct for malaria elimination activities by identifying foci of residual transmission not captured by case-based passive surveillance or cross-sectional surveys. Where funding is available, molecular analysis of *Plasmodium* isolates from pregnant women may characterize genetic signatures of malaria transmission intensity, anti-malarial resistance, and detection of deletions of antigens targeted by RDTs [22, 23] in a way that is easily obtained yet representative of the general population.

### How results will be used

Routine malaria testing of all ANC1 attendees irrespective of symptoms potentially improves pregnancy outcomes by testing and treating women with asymptomatic but detectable parasitaemia. The results generated will add to existing knowledge and evidence that supports use of women attending ANC1 as a pragmatic sentinel (Additional file 1) population for malaria surveillance. The present study will continue to expand the evidence base generated by studies characterizing the relationship between malaria infection in pregnant women and in children in the same communities [9] across new locations and various transmission settings. Geospatial and temporal mapping of infection prevalence by village and/or health facility may also reveal specific hot spots [11].

### Challenges

ANC1-based surveillance requires strengthening systems for routine data collection and changing data management and reporting procedures, as ANC registers would need to include additional fields such as questions on insecticide-treated net use and care-seeking, which are not typically recorded. Routinely aggregated data such as women’s age and gravidity would need to be included at an individual level to optimize analysis of prevalence trends. To be most useful, data would need to be collected and reviewed in near real-time, which can be a challenge. Scaling ANC1-based surveillance requires investment in the capacity of midwives, which would involve training on revised policy guidelines to test and treat all women, increasing the number of midwives to support malaria testing, and/or identification of other cadres of workers who can assist in collecting this information. While theoretically, universal malaria testing at ANC1 could increase the duration of the appointment or disrupt patient flow, studies assessing the feasibility have not found this to be the case [24–27]. Increased funding would be required to ensure that health facilities have sufficient RDTs for universal testing and to avoid stockouts of antimalarial treatments. Funding would also need to support modification of HMISs to distinguish between positive infections in women being tested routinely at ANC1 from symptomatic pregnant women tested during unscheduled visits or those later in pregnancy.

### Study limitations

Using ANC1 for malaria surveillance depends on the extent to which pregnant women attend public ANC clinics, which is affected by nonattendance or attendance at private ANC clinics, among other factors. In countries where attendance at private ANC clinics is high, or if variations exist among rural and urban areas, underrepresentation of private clinics in the surveillance system could introduce selection bias if populations of pregnant women attending private and public ANC clinics differ with regard to malaria exposure [15]. Because private facilities are more commonly located in urban settings, where malaria risk is generally lower, ANC1 data may not be fully representative of the population. Similarly, areas with poor access to health facilities in general may be more likely to be rural and have higher malaria prevalence; this could induce bias if women who present for care are in the areas with lower prevalence. Additionally, bias may arise at screening, particularly if RDTs are limited, if symptomatic women are preferentially tested. Finally, while health facilities have defined catchment areas, people may, to varying extent, choose to seek care outside of their defined health facility. Defining catchment areas strictly is a complex task for many surveillance indicators [28], including malaria case metrics [29]. As data are aggregated upward to larger areas, i.e., districts rather than specific health facility catchments, the effect of people’s movement is likely diminished. To better account for movement of people outside of their designated catchment areas, one could collect data on where people reside; this was done in Zambia where it was recognized that many of the women seeking care in border facilities might have crossed over from Mozambique, allowing for exclusion of these women from the comparison with the household survey data.

## Conclusions

ANC1-based surveillance offers the potential of a lower-cost, more granular, and potentially more representative measure of community malaria burden and other indicators typically sought through cross-sectional surveys. While there are numerous potential benefits of this method of malaria surveillance, it needs to be rigorously validated across a range of settings, particularly with respect to measuring coverage indicators for malaria control measures. As with many routine data collection efforts, post-validation challenges would remain, not the least of which would be ensuring adequate stock of commodities, expanded training requirements, register modifications, and policy changes.

## Supplementary Information

**Additional file 1: **ANC1 Surveillance Questions.

## Data Availability

The datasets used and/or analysed during the current study are available from the corresponding author on reasonable request.

## Abbreviations

ANC: Antenatal care
ANC1: First antenatal care visit
DHIS2: District Health Information Software 2
HMIS: Health management information system
IPTp: Intermittent preventive treatment in pregnancy
RDT: Rapid diagnostic test
