# Supplementary material for: Using antenatal care as a platform for malaria surveillance data collection: study protocol
Source: Malar J. 2023 Mar 17;22:99. doi: 10.1186/s12936-023-04521-6 (PMC10022568; doi:10.1186/s12936-023-04521-6)
Supplement: Supplementary file 1 — Additional file 1: ANC1 Surveillance Questions. [file 12936_2023_4521_MOESM1_ESM.docx]

# **Additional file 1: ANC1 SURVEILLANCE QUESTIONS**

| # | Question | **Response** | Skip pattern |
| --- | --- | --- | --- |
| 0.1 | Indicate the name of the facility |  |  |
| 0.2 | Indicate the village where the woman resides |  |  |
| The ministry of health is piloting a series of questions to inform their malaria control program. You may choose to answer or not answer any or all of these questions with no penalties to you. May I proceed with the questions? | | | |
| 0.3 | Did the woman agree to answer the questions? | **YES………………………...1 NO………………………….2** | If “1” then skip to 0D  If “2” then skip to 0C and END |
| 0.4 | Why did the woman not agree? | **The woman was not interested…...1 The woman does not have time.…….2**  **Reason was not specified……….…….3** |  |
| 0.5 | Did the woman accept to take a malaria rapid diagnostic test (RDT)? | **YES………………………...1 NO………………………….2** | If “2” continue to 1 |
| PROCEED TO ASK THE WOMAN | | | |
| 0.6 | Have you had any fever episodes in the past 2 days? | **YES………………………...1 NO………………………….2** |  |
|  | How many people usually live in your household? | **Number of people \|____\|_____\|** |  |
|  | How many insecticide treated mosquito nets (LLIN) does your household have? | **Number of nets \|____\|_____\|** | If "0" then skip to 7 |
|  | Did you sleep under an LLIN last night? | **YES………………………...1 NO………………………….2** |  |
|  | How many children under 5 slept in your household last night? | **Number of children \|____\|_____\|** | If "0" then skip to 7 |
|  | How many of those children slept under a net last night? | **Number of children \|____\|_____\|** |  |
|  | Is this your first pregnancy? | **YES………………………...1 NO………………………….2** | If YES then END |
|  | How many prior pregnancies have you had? | **Number of pregnancies \|____\|_____\|** |  |
|  | How many children under 5 do you have? | **NUMBER OF CHILDREN \|____\|____\|** | If "0" then END |
|  | Have any of your children who are under 5 years old been ill with a fever at any time in the last 2 weeks? | **YES………………………...1 NO………………………….2** | If YES then continue to 9B  If NO then END |
| 9B. | How many of your children under 5 years old have been ill with a fever at any time in the last 2 weeks? | **Number of children \|____\|_____\|** | Continue to question 10 and repeat for EACH child |
|  | For each child aged < 5years with fever in the past 2 weeks: at any time during the illness, did you seek any advice or treatment for the illness from any source? | **YES………………………...1 NO………………………….2** | If NO then END |
|  | Where did you seek treatment? | **GOVERNMENT HOSPITAL…………………...01**  **GOVERNMENT HEALTH CENTER………….02**  **GOVERNMENT HEALTH POST………………03**  **MOBILE CLINIC ………………………………04**  **FIELDWORKER ………………………………05**  **OTHER PUBLIC SECTOR…………………06**  **PRIVATE HOSPITAL/CLINIC……………07**  **PHARMACY ……………………………………08**  **PRIVATE DOCTOR ……………………….…09**  **FIELDWORKER ………………………………10**  **OTHER PRIVATE MEDICAL SECTOR……..11**  **SHOP …………………………………………………12**  **TRADITIONAL PRACTITIONER ……………13**  **MARKET ……………………………………………14**  **ITINERANT DRUG SELLER……………………15**  **OTHER………………………………………………97** |  |
|  | How many days after the illness began did you first seek advice or treatment for this child? | **NUMBER OF DAYS** |  |
|  | Did this child have blood taken from his/ her finger or heel for malaria testing? | **YES………………………...1 NO………………………….2** |  |
|  | At any time during the illness, did this child take any drugs for the illness? | **YES………………………...1 NO………………………….2** | - Continue - END |
|  | What drugs did this child take? | **ARTEMISININ COMBINATION THERAPY**  **(ACT)…………………………01**  **SP/FANSIDAR ………………………….…02**  **CHLOROQUINE……………………………03**  **AMODIAQUINE……………………………04**  **QUININE……………………………………..05**  **OTHER (specify) __________________06**  **ARTESUNATE INJECTION ………….…...07**  **QUININE INJECTION………………………08**  **RECTAL ARTESUNATE……………….……09**  **ANTIBIOTIC DRUGS (ORAL) PILL/SYRUP ……………………………..10**  **ANTIBIOTIC INJECTION/IV……………11**  **DON'T KNOW………………………………12** |  |
|  | MATERNAL RDT RESULT | **NEGATIVE ………………………………1**  **POSITIVE …………….…………………2**  **INVALID….………………..………………3** |  |
